# ERCC1 Expression Analysis to Guide Therapy in Non-Small Cell Lung Cancer

**DOI:** 10.1371/currents.RRN1202

**Published:** 2010-12-06

**Authors:** Diane Allingham-Hawkins, Andrew Lea, Susan Levine

**Affiliations:** Hayes, Inc.

## Abstract

Worldwide, lung cancer accounts for approximately 1 million deaths each year, making it the most common cause of cancer-related mortality. Non-small cell lung cancer (NSCLC) accounts for approximately 85% of lung cancer cases and is often associated with a relatively poor prognosis. The majority of NSCLC patients present with advanced disease and have an average 5-year survival rate of 5%. Currently, the standard of care for NSCLC includes treatment with a platinum-based chemotherapy regimen. However, not all patients benefit equally from such treatment. Therefore, recent pharmacogenomic studies have been performed in order to identify specific biomarkers that may allow for patient-tailored treatment strategies. One such biomarker is expression of the excision repair cross-complementation group 1 protein, ERCC1.

## **Clinical Scenario**

Lung cancer, the most common cause of cancer-related death among both men and women, may be classified as either small cell or non-small cell[1] [2]. Non-small cell lung cancer (NSCLC) is more common, accounting for approximately 85% of lung cancer cases[3]. Most patients with NSCLC present with advanced disease and have a relatively poor prognosis[3]. Advanced NSCLC is associated with a 5% survival rate at 5 years, while the overall 5-year survival for all stages is only 15%[1] [4]. NSCLC tends to be less chemosensitive than small cell lung cancer and is typically treated with platinum-based chemotherapy regimens[1] [3] [4] [5]. Overall, response rates for inoperable NSCLC range from 30% to 60% when treated with a platinum-based chemotherapeutic agent (such as cisplatin or carboplatin) combined with gemcitabine, vinorelbine, or taxane[1] [6]. In addition, adjuvant chemotherapy using a platinum-based protocol improves overall survival by up to 15%, depending on the stage of the tumor[6]. While platinum-based chemotherapy regimens are a standard treatment for NSCLC, the NSCLC patient population is quite heterogeneous and includes individuals with varying degrees of sensitivity to this type of treatment[6] [7] [8]. In general, the goal of pharmacogenomic testing in oncology is to utilize the molecular characteristics of a tumor or the genetic traits of an individual to improve patient outcomes[7]. Such characteristics, or biomarkers, may be of prognostic value, indicating which patients have a better or worse prognosis based on molecular profile, or may be used in a predictive capacity, indicating which patients may or may not benefit from particular treatment modalities[4] [7] [9]. One of the biomarkers currently being examined in NSCLC patients is expression of the gene encoding the excision repair cross-complementation group 1 protein, ERCC1[4]. The ERCC1 enzyme plays a key role in the nucleotide excision repair (NER) pathway, and also removes cisplatin-induced DNA adducts (these consist of covalent bonds between DNA and a carcinogen)[10]. ERCC1 expression levels, measured by assessing messenger RNA (mRNA) or protein levels in tumor cells, may have both prognostic and predictive value to NSCLC patients[7]. Recent studies have suggested that higher levels of ERCC1 expression may be associated with a better prognosis overall for patients with NSCLC[1] [7]. Conversely, other studies have suggested that higher levels of ERCC1 expression may be associated with an increased resistance to platinum-based chemotherapeutic agents[1] [3] [7] [11] [12] [13] [14]. The potential patient population for ERCC1 gene expression analysis is all patients with NSCLC that are being considered for treatment with a platinum-based chemotherapeutic regimen[6] [7] [8] [15].

## **Test Description**

ERCC1 gene expression analysis for NSCLC is performed clinically using immunohistochemistry (IHC)[2]. The antibody 8F1 (Neomarkers; Fremont, CA), which recognizes the ERCC1 protein, is used to assess ERCC1 levels within tumor cells present in tissue sections obtained from lung biopsy or tumor resection. Within the United States, this test is offered by Genzyme Genetics (Cambridge, MA)[15] [16] [17] and Quest Diagnostics (Madison, NJ)[18] [19]. According to information provided by Genzyme Genetics, the levels of ERCC1 are based on the intensity of staining when compared with internal controls, namely, lymphocytes and stromal cells within the same sample[17]. A result of 0 indicates that there is no reactivity to the antibody in tumor cells. A result of 1+ indicates that the reactivity in tumor cells is less than that seen in control cells, while a result of 2+ indicates that the reactivity is similar in both sets of cells. Finally, a result of 3+ indicates that the reactivity is greater in tumor cells than control cells. Subsequently, the result is said to be “positive” if there is 3+ staining in more than 10% of tumor cells. The result is said to be “negative” if there is 0, 1+, or 2+ staining in tumor cells, or if there is 3+ staining in less than 10% of tumor cells[17]. No details are provided on the ERCC1 expression test available from Quest Diagnostics, other than that it is available both with and without interpretation[18] [19].

## **Public Health Importance**

Lung cancer is the leading cause of cancer-related mortality in the United States and NSCLC accounts for approximately 85% of all lung cancers. Although platinum-based chemotherapy are a standard treatment for NSCLC, not all patients with NSCLC respond to this type of treatment. Therefore, the availability of a biomarker that effectively identifies those patients most likely to respond to platinum-based chemotherapy is desirable for two reasons: first, those who are likely to respond can be treated using platinum-based chemotherapy regimens and; second, those who are not likely to respond can be treated using other regimens as a first-line treatment[6] [7] [8].

## **Published Reviews, Recommendations and Guidelines** 

**Systematic evidence reviews**

None identified.

**Recommendations by independent group**

None identified.

**Guidelines by professional groups**

National Comprehensive Cancer Network (NCCN) -The NCCN’s clinical practice guideline regarding NSCLC includes information about the role of ERCC1 as both a prognostic and predictive biomarker for NSCLC. However, the guidelines do not include recommendations about the use of ERCC1 testing in NSCLC patients[20].

## **Search Strategy**

A literature search of MEDLINE and EMBASE was completed on September 18, 2010, using the search terms (*excision repair cross-complementation group 1* OR *ERCC1*) AND (*non-small cell lung cancer* OR *NSCLC*). After limiting to English language, human, and published since January 1, 1996, this search yielded 122 citations. Citations from relevant references were also reviewed and included as appropriate. 

## **Evidence Overview** 

***Analytic Validity*:**Test accuracy and reliability in measuring ERCC1 expression (analytic sensitivity and specificity).

ERCC1 testing is performed by IHC using an antibody to detect the ERCC1 protein. Two studies evaluated the specificity of the 8F1 ERCC1 antibody and yielded conflicting results:

- Niedernhofer and colleagues (2007) compared the performance of 8F1 with that of a second commercially available antibody, FL-297. Test samples included normal human fibroblasts (positive control) and cells from patients with inherited sequence variants in the *ERCC1* and xeroderma pigmentosum complementation group F (*XPF*) genes. Using the FL-297 antibody, immunoblotting of cell lysates revealed a single band of the expected molecular weight in normal fibroblasts, but reduced levels of the protein in the patient cells. Using the 8F1 antibody, however, immunoblotting of cell lysates from normal fibroblasts revealed two separate bands: a band of unknown origin and a band corresponding to the ERCC1 protein. In the patient cell lines, only a single band was seen, which the authors state represented a protein of unknown origin that is cross-reacting to the 8F1 antibody. In a second experiment, normal fibroblasts and cells from the patient with an *XPF* gene variant were differentially labeled and co-cultured. Immunostaining revealed that the FL-297 antibody was reactive in the normal fibroblasts, but not in the cells from the patient. In contrast, the 8F1 antibody yielded nuclear staining in all cells. Immunostaining of cells from the patient with an *ERCC1* gene variant was not reported. The authors concluded that these results suggest that the 8F1 antibody is not specific for the ERCC1 protein and cannot discriminate between ERCC1-positive and ERCC1-deficient cells. Data confirming decreased expression of ERCC1 in the patient cell lines were not included in this study[21].
- Olaussen and colleagues (2007) further studied the specificity of the 8F1 antibody in response to the claim by Niedernhofer and colleagues (2007) that this antibody may not be specific for ERCC1. Several complementary approaches using small interfering RNAs (siRNAs) were utilized. First, siRNAs designed to block *ERCC1* expression were used to deplete two epithelial carcinoma cell lines (including one NSCLC cell line) of ERCC1 protein. Using the 8F1 antibody, immunoblotting detected a single band of the expected molecular weight in each cell line. After treatment with the siRNA, this band disappeared. In a second experiment, cell pellets from treated and untreated cell lines were fixed and embedded using the same methods used for resected lung tumors. Subsequently, IHC was performed using the 8F1 antibody. Nuclear ERCC1 staining was clearly evident in the untreated control cells; however, this staining was absent in cells that were transfected with the siRNA. The authors concluded that these results suggest that 8F1 is specific when used for the immunohistochemical detection of ERCC1 in NSCLC samples[21] [22].

Two studies evaluated the importance of specimen type for ERCC1 expression testing:

- Taillade and colleagues (2007) evaluated the expression levels of ERCC1 (in addition to four other biomarkers for NSCLC) in both bronchial biopsies and surgical specimens (i.e., resected tumors) in 34 patients. The 8F1 antibody was used for IHC and two separate pathologists scored each sample blindly. Results were dichotomized into negative and positive based on a cutoff of 10% ERCC1 antibody reactivity within tumor cells (i.e., a positive result indicated that at least 10% of cells demonstrated a reactivity to the ERCC1 antibody). Seven samples were found to lack a valid internal control and were excluded from the analysis. The discordance in scoring between the two pathologists (indicated by a discrepancy > 20% in staining percentage) was 7%. Overall, when comparing the results obtained from biopsies and surgical specimens, a statistically significant correlation was found (correlation coefficient r=0.83; P<0.0001). With the cutoff of 10%, 8 (30%) biopsies and 11 (41%) resected tumors were found to be positive for ERCC1. Thus, the discordance between specimen types, when examining positive versus negative results, was 9% (95% confidence interval [CI], 0% to 18.4%). The authors concluded the above results suggest that, while there is a strong correlation between ERCC1 levels in bronchial biopsies and resected tumor specimens, discordance is evident for different specimen types in some cases[23]
- Gomez-Roca and colleagues (2009) assessed the levels of ERCC1 in primary NSCLC tumor specimens  compared with the levels found in corresponding metastases. IHC using the 8F1 antibody was performed in 49 NSCLC patients who had both types of test specimens available. Using an H score (semiquantitative scores calculated by summing the products of staining intensities and distributions) of 1 to classify the ERCC1 expression, 18 primary tumors and 26 metastatic lesions were found to be ERCC1 positive. However, there was a significantly higher level of ERCC1 expression in metastases than in primary tumors (P=0.04). ERCC1 expression was at least five times greater in metastatic lesions (especially brain metastases) than in corresponding tumors. ERCC1 status was discordant in 20 of the 49 (41%) patients. The authors concluded that these results suggest that it may be appropriate to examine ERCC1 levels in both primary tumors and metastatic lesions prior to deciding on a treatment regimen[24].

***Clinical Validity*:**Test accuracy and reliability in identifying patients with an improved prognosis and/or likely to respond to platinum-based chemotherapy regimens (predictive value). 

ERCC1 expression levels have been investigated as both a prognostic marker and as a predictive marker regarding treatment. 

Seven studies were identified that investigated the correlation between ERCC1 expression levels and prognosis (see Table 1). Five of these studies reported a statistically significant difference in overall survival with ERCC1-positive patients experiencing significantly longer survival than ERCC1-negative patients[25] [26] [27] [10] [28].Two studies, however, reported no significant difference between ERCC1-positive and ERCC1-negative patients with respect to survival[29] [30].The patient populations examined in these two studies varied slightly from the other studies. While the others included patients with stage I to stage III NSCLC, Okuda and colleagues (2008) included patients with any stage NSCLC[30] and Bartolucci and colleagues (2009) included only patients with stage IB to IIB NSCLC[29]. In addition, the categorization of patients used by Bartolucci and colleagues (2009) differed from the other six studies. In this study, patients were categorized into low, intermediate, and high ERCC1 expression groups[29]. In contrast, all other studies used the median expression level as a cutoff for high versus low (or positive versus negative) ERCC1 expression[25] [26] [27] [10] [28] [30]. Whether or not these differences in study design contributed to the conflicting results is unclear.

**Table 1. Studies Evaluating ERCC1 as a Prognostic Biomarker in Patients with NSCLC.**

**Key:** AQUA, automated quantitative analysis; CI, confidence interval; ERCC1, excision repair cross-complementation group 1; HR, hazard ratio; IHC, immunohistochemistry; NSCLC, non-small cell lung cancer; postop, postoperative(ly); pt(s), patient(s); ref, reference; rRNA, ribosomal ribonucleic acid; RT-PCR, reverse transcription polymerase chain reaction

**Table:** 

**Reference**	**Population**	**ERCC1 Analysis Methodology**	**Results**
Simon and colleagues (2005)[28]	51 pts w/ stage IA-IIIB, resected NSCLC (4 had postop radiation and 1 had radiation plus chemotherapy)	RT-PCR (ref: 18S rRNA gene expression*); median expression level used as cutoff for high vs low expression	Median overall survival was 94.6 mos for pts w/ high ERCC1 levels and 35.5 mos for pts w/low ERCC1 levels (P=0.01); HR for death in those w/ high ERCC1 levels was 0.242 (95% CI, 0.076-0.775; P=0.0168)
Olaussen and colleagues (2006)[10]	761 pts w/ stage I-III NSCLC	IHC; median H score used as cutoff for negative vs positive expression†	Overall survival was 46% (95% CI, 37% to 55%) for ERCC1-positive pts and 39% (95% CI, 32% to 47%) for ERCC1-negative pts; adjusted HR for death in ERCC1-positive pts was 0.66 (95% CI, 0.49-0.90; P=0.009)
Zheng and colleagues (2007)[25]	41 pts w/ stage I-III, resected NSCLC	IHC (w/ AQUA); median expression level used as cutoff for high vs low expression	Improved overall survival in pts w/ high levels of ERCC1 (median survival not provided) (P=0.01)
Lee and colleagues (2008)[26]	130 pts w/ stage I-III, resected NSCLC	IHC; median H score used as cutoff for negative vs positive expression†	HR for death in ERCC1-positive pts was 0.598 (95% CI, 0.357-1.001; P=0.051)
Okuda and colleagues (2008)[30]	59 pts w/ stage I-IV, resected NSCLC	IHC; median H score used as cutoff for negative vs positive expression†	No significant difference in survival was noted between ERCC1-positive and ERCC1-negative pts (P=0.2494)
Bartolucci and colleagues (2009)[29]	54 pts w/ stage IB-IIB, resected NSCLC	RT-PCR (ref: beta-actin gene expression*); pts categorized into low, intermediate, and high ERCC1 expression groups	No significant correlation between ERCC1 expression level and survival (overall or disease-free) was identified
Koh and colleagues (2010)[27]	136 pts w/ stage I-IIIb, resected NSCLC	IHC; median H score used as cutoff for negative vs positive expression†	Median overall survival was 108 mos for ERCC1-positive pts and 47 mos for ERCC1-negative pts (P=0.064) Median relapse-free survival was 54 mos for ERCC1-positive pts and 32 mos for ERCC1-negative pts (P=0.526)

* ERCC1 expression is typically normalized by comparing with expression of a constitutively expressed housekeeping gene (such as the genes encoding 18S rRNA or beta-actin).

† H scores are semiquantitative scores calculated by summing the products of staining intensities and distributions.

Seventeen studies were identified that investigated the predictive value of ERCC1 expression levels with respect to response to treatment with platinum-based chemotherapy agents (see Table 2). Eleven studies reported a statistically significant increase in overall survival in ERCC1-negative patients treated with platinum-based chemotherapy regimens compared with ERCC1-positive patients[31] [32] [33] [34] [35] [36] [37] [38] [39] [40] [41]. Several additional studies revealed a trend for improved overall survival in patients with low levels of ERCC1, although their findings were not statistically significant[42] [43]. Four remaining studies reported no difference in survival[44] [45] [46] [47]. While there appears to be a clear relationship between ERCC1 status and survival in patients treated with platinum-based chemotherapy, most of the studies described above found no significant correlation between response rate and ERCC1 expression[35] [37] [38] [39] [41] [45]. Finally, it is of note that a single study by Booten and colleagues reported findings that seem contradictory to the others.[45] In this study, the median survival was longer in patients with high levels of ERCC1 expression than in those with low levels (415 days for high ERCC1 vs 327 for low ERCC1; P=0.801), although this finding was not statistically significant. One difference in study design between this study and the others that utilized reverse transcription polymerase chain reaction (RT-PCR) was the use of an NSCLC control gene (as opposed to a constitutively expressed housekeeping gene) as the reference gene for calculating expression levels. The authors of this analysis also included an additional calculation to correct for differences in amplification efficiency[45].

**Table 2. Studies Evaluating ERCC1 as a Predictive Biomarker in patients with NSCLC.**

**Key: **APPBP2, amyloid beta precursor protein (cytoplasmic tail) binding protein 2; CI, confidence interval; ERCC1, excision repair cross-complementation group 1; GAPDH, glyceraldehyde-3-phosphate dehydrogenase; HR, hazard ratio; IHC, immunohistochemistry; NSCLC, non-small cell lung cancer; pt(s), patient(s); ref, reference; RT-PCR, reverse transcription polymerase chain reaction.

**Table:** 

**Reference**	**Population**	**Treatment**	**ERCC1 Analysis Methodology**	**Results Regarding Treatment Response and Survival**
Lord and colleagues (2002)[41]	56 pts w/ stage IIIb or IV NSCLC (30 adenocarcinomas, 20 squamous cell carcinomas, 4 large cell carcinomas, and 2 unspecified)	Gemcitabine + cisplatin	RT-PCR (ref: beta-actin gene expression*); median expression level used as cutoff for high vs low expression	**Median Overall Survival:** 61.6 wks (95% CI, 42.4-80.7) for low ERCC1 vs 20.4 wks (95% CI, 6.9-33.9) for high ERCC1 (P=0.009); HR for death in pts w/ low ERCC1 expression was 0.32 (95% CI, 0.14-0.71; P=0.005) **Median Response Rate:** 52% for low ERCC1 vs 36.4% for high ERCC1 (P=0.38)
Rosell and colleagues (2004)[43]	81 pts w/ stage IIIb or IV NSCLC (39 adenocarcinomas, 39 squamous cell carcinomas, 3 large cell carcinomas)	Gemcitabine + cisplatin (GC; n=21); gemcitabine + cisplatin + vinorelbine (GCV; n=31); gemcitabine + vinorelbine + ifosfamide (GVI; n=29)	RT-PCR (ref: beta-actin gene expression*); median expression level used as cutoff for high vs low expression	No significant correlation between survival and ERCC1 expression was noted in the GCV or GVI treatment arms. **GC Treatment Arm:Median OverallSurvival:** 13.7 wks (95% CI, 9.6-17.8) for low ERCC1 vs 9.5 wks (95% CI, 6.3-12.8) for high ERCC1 (P=0.19) **Median Time to Progression: **8.4 wks (95% CI, 4.5-12.2) for low ERCC1 vs 5.1 wks (95% CI, 1-9.3) for high ERCC1 (P=0.07)
Rosell and colleagues (2004)[47]	67 pts w/ stage IIB-IIIB NSCLC (26 adenocarcinomas, 29 squamous cell carcinomas, 11 large cell carcinomas, and 1 other)	Gemcitabine + cisplatin (n=63) or gemcitabine + carboplatin (n=4)	RT-PCR (ref: beta-actin gene expression*); expression levels divided into quartiles	**Overall Survival:** The relative risk of death was 1.51 (95% CI, 0.55-4.10; P=0.422) for pts w/ expression in the lowest quartile, when compared w/ expression in the top quartile
Ceppi and colleagues (2006)[32]	61 pts w/ stage III or IV NSCLC (33 adenocarcinomas, 17 squamous cell carcinomas, 11 large cell carcinomas)	Gemcitabine + cisplatin (GC; n=43) or gemcitabine alone (G; n=18)	RT-PCR (ref: beta-actin gene expression*); median expression level used as cutoff for high vs low expression	**All Patients Median Overall Survival:** 17.3 mos for low ERCC1 vs 10.9 mos for high ERCC1 (P=0.0032); HR for death in pts w/ high ERCC1 expression was 3.43 (95% CI, 1.71-6.86; P=0.0003) **GC Treatment Arm Median Overall Survival: **23 mos for low ERCC1 vs 12.4 mos for high ERCC1 (logrank 10.3; P=0.001) No significant correlation between survival and ERCC1 expression was noted in the gemcitabine alone (G) treatment arm.
Olaussen and colleagues (2006)[10]	761 pts w/ stage I-III NSCLC (242 adenocarcinomas, 426 squamous cell carcinomas, and 93 unspecified)	Cisplatin + etoposide or vinca alkaloid (n=389) or observation only (n=372)	IHC (8F1 antibody); median H score used as cutoff for negative vs positive expression†	**Overall Survival: **Adjusted HR for death in treated pts w/ ERCC1-negative tumors was 0.65 (95% CI, 0.50-0.86; P=0.002), when compared w/ untreated pts. Pts with ERCC1-positive tumors did not benefit from cisplatin treatment as the adjusted HR for death was 1.14 (95% CI, 0.84 to 1.55; P=0.40).
Azuma and colleagues (2007)[39]	67 pts w/ stage IAIIIB NSCLC (53 adenocarcinomas and 14 squamous cell carcinomas)	Cisplatin doublet (n=21); carboplatin doublet (n=46)	IHC (8F1 antibody); 25% staining used as cutoff for negative vs positive expression	**Response Rate:** 28% for ERCC1 positive and 29% for ERCC1 negative **Median Progression-free Survival: **44 wks for ERCC1 negative vs 26 wks for ERCC positive (P=0.007); HR, 1.37 (95% CI, 1.06-1.76; P=0.014) **Median Overall Survival:** 73 wks for ERCC1 negative vs 44 wks for ERCC positive (P=0.001); HR, 1.65 (95% CI, 1.21-2.28; P=0.002)
Booton and colleagues (2007)[45]	66 pts w/ stage III or IV NSCLC (17 adenocarcinomas, 29 squamous cell carcinomas, 3 large cell carcinomas, and 17 unspecified)	Cisplatin triplet or carboplatin doublet	RT-PCR (ref: APPBP2 gene expression*); specific cutoff for determining high vs low expression was unclear	**Response Rate:** 36% for high ERCC1 and 28% for low ERCC1 (P=0.794) **Median Survival:** 415 days (95% CI, 197-633) for high ERCC1 vs 327 (95% CI, 211-433) for low ERCC1 (P=0.801); HR for death 0.96 (95% CI, 0.919-1.004; P=0.08) for high ERCC1
Hsu and colleagues (2007)[46]	30 pts w/ stage IIIB or IV NSCLC (majority of pts had adenocarcinomas or squamous cell carcinomas)	Cisplatin + gemcitabine or gemcitabine + epirubicin	IHC (8F1 antibody); 10% staining used as cutoff for negative vs positive expression	No significant difference was noted in response rate, time-to-treatment-failure, or overall survival
Fujii and colleagues (2008)[44]	35 pts w/ stage IIIa or IIIb NSCLC (19 adenocarcinomas, 11 squamous cell carcinomas, and 5 other types)	Cisplatin + irinotecan (n=15) or cisplatin + docetaxel + radiation (n=20)	IHC (8F1 antibody); median percentage of stained cells used as cutoff for negative vs positive expression	**C** **hemotherapy Group Response Rate:** 100% for ERCC1 negative and 42.9% for ERCC1 positive (P=0.013) **Chemoradiotherapy GroupResponse Rate:** 57.1% for ERCC1 negative and 46.2% for ERCC1 positive (P=1.0) No significant difference in overall or disease-free survival was noted for either group.
Hwang and colleagues (2008)[33]	68 pts w/ stage IIIA, N2-positive NSCLC (41 adenocarcinomas, 26 squamous cell carcinomas, and 1 unspecified)	Cisplatin doublet or carboplatin doublet	IHC (8F1 antibody); median H score used as cutoff for negative vs positive expression†	**Response Rate:** Complete or partial response in 81% of ERCC1-positive and 87% of ERCC1-negative tumors (P=0.515); downstaging in 48% of ERCC1-positive and 65% of ERCC1-negative tumors (P=0.171)**Median Overall Survival:** 26.0 mos for ERCC1 positive vs 89.2 mos for ERCC1 negative (P=0.014) **Median Progression-free Survival:** 15.9 mos for ERCC1 positive vs 29.5 mos for ERCC1 negative (P=0.062)
Okuda and colleagues (2008)[40]	90 pts w/ stage I-IV NSCLC (44 adenocarcinomas, 33 squamous cell carcinomas, 6 large cell carcinomas, and 7 adenosquamous cell carcinomas)	Cisplatin doublet or carboplatin doublet	IHC (8F1 antibody); median H score used as cutoff for negative vs positive expression†	**Median Overall Survival:** 37.6 mos for ERCC1 positive vs 60.8 mos for ERCC1 negative (P=0.0068); HR for death in ERCC1-positive cases was 2.181 (95% CI, 1.167-4.076; P=0.0145)
Azuma and colleagues (2009)[42]	45 pts w/ stage IAIIIB NSCLC (36 adenocarcinomas and 9 squamous cell carcinomas)	Carboplatin + paclitaxel	IHC (8F1 antibody); 10% staining used as cutoff for negative vs positive expression	**Response Rate: **Complete or partial response in 20% of ERCC1-positive and 28% of ERCC1-negative tumors (P=0.729) **Median Overall Survival: **56 wks for ERCC1 positive vs 102 wks for ERCC1 negative (P=0.01) **Median Progression-free Survival:** 28 wks for ERCC1 positive vs 44 wks for ERCC1 negative (P=0.046)
Azuma and colleagues (2009)[38]	34 pts w/ stage IIb-IIIb NSCLC (16 adenocarcinomas, 17 squamous cell carcinomas, and 1 other)	Cisplatin + docetaxel	IHC (8F1 antibody); 10% staining used as cutoff for negative vs positive expression	**Response Rate:** Complete or partial response in 37.5% of ERCC1-positive and 83% of ERCC1-negative tumors (P=0.012) **Median Overall Survival: **50.5 wks for ERCC1 positive vs 171 wks for ERCC1 negative (P=0.208) **Median Progression-free Survival:** 36 wks for ERCC1 positive vs 62.5 wks for ERCC1 negative (P=0.009)
Ikeda and colleagues (2009)[34]	40 pts w/ stage III or IV NSCLC (13 adenocarcinomas, 23 squamous cell carcinomas, and 4 large cell carcinomas)	Carboplatin + paclitaxel	IHC (8F1 antibody); 10% staining used as cutoff for negative vs positive expression	**Average Overall Survival: **398 days for ERCC1 positive vs 932 days for ERCC1 negative (P=0.014); HR for death in ERCC1-positive pts was 3.485 (95% CI, 1.123-10.818; P=0.031)
Lee and colleagues (2009)[26]	50 pts w/ stage IIIb or IV NSCLC (34 adenocarcinomas and 16 squamous cell carcinomas)	Cisplatin doublet or carboplatin doublet	IHC (ERCC1 Ab-2‡ [Neomarkers]); median H score used as cutoff for high vs low expression†	**Response Rate:** Complete or partial response in 39% of high ERCC1 and 32% of low ERCC1 tumors (P=0.768) **Median Overall Survival:** 8 mos for high ERCC1 and 11 mos for low ERCC1 tumors (P=0.055); HR for death in pts w/ high ERCC1 levels was 3.156 (95% CI, 1.54-6.46; P=0.002)
Li and colleagues (2009)[36]	60 pts w/ stage IBIIIA NSCLC (35 adenocarcinomas and 25 squamous cell carcinomas)	Cisplatin doublet	RT-PCR (ref: GAPDH gene expression*); median expression level used as cutoff	**Overall Survival:** High levels of ERCC1 correlated w/ poor overall survival (P=0.012); HR for death in pts w/ high ERCC1 levels (using ERCC1 expression as a continuous variable) was 2.28 (95% CI, 1.09-3.66; P=0.009) **Tumor-free Survival: **ERCC1 expression was not significantly correlated w/ tumor-free survival (P=0.123)
Ota and colleagues (2009)[37]	156 pts w/ stage IV NSCLC (100 adenocarcinomas and 56 other)	Cisplatin doublet or carboplatin doublet	IHC (8F1 antibody); 10% staining used as cutoff for negative vs positive expression	**Response Rate: **Complete or partial response in 26% of ERCC1-positive and 27% of ERCC1-negative tumors (P>0.99) **Median Overall Survival:** 237 days for ERCC1 positive vs 453 days for ERCC1 negative (P=0.03) **Median Progression-free Survival:** 148 days for ERCC1 positive vs 187 days for ERCC1 negative (P=0.06)

* ERCC1 expression is typically normalized by comparing with expression of a constitutively expressed housekeeping gene (such as the genes encoding 18S rRNA or beta-actin).

† H scores are semiquantitative scores calculated by summing the products of staining intensities and distributions.

‡ It is unclear if this is the 8F1 antibody.

***Clinical Utility*: **Net benefit of test in improving health outcomes.

Two clinical trials were identified that investigated the impact of ERCC1 expression testing on patient outcomes:

- A prospective phase II clinical trial investigated the impact of ERCC1 and ribonucleotide reductase M1 subunit (RRM1), another biomarker that has been associated with treatment response in NSCLC, expression testing on patient outcomes in 55 patients with advanced NSCLC (stage IIIb or stage IV). An additional 5 patients were eligible for the trial, but did not have sufficient tumor material available for analysis. Fifty-three of the patients subsequently entered one of four treatment arms. Patients with low levels of both ERCC1 and RRM1 were treated with carboplatin and gemcitabine (n=12). Patients with high levels of both ERCC1 and RRM1 were treated with docetaxel and vinorelbine (n=14). Patients with high ERCC1 and low RRM1 were treated with gemcitabine and docetaxel (n=20). Patients with low ERCC1 and high RRM1 were treated with carboplatin and docetaxel (n=7). A total of 43 patients received between three and six cycles of treatment; 2 patients only received one cycle of treatment (1 of whom was not evaluated for treatment response). Overall, a partial remission was obtained in 23 (44%) patients; the median overall survival was 13.3 months (95% CI, 11.5 months to < 24 months), and the median progression-free survival was 6.6 months (95% CI, 4.7 to 8.8 months). There was no significant correlation between gene expression and treatment response (P=0.33 for ERCC1; P=0.28 for RRM1), between gene expression and overall survival (HR, 1.0 and P=0.19 for ERCC1; HR, 0.996 and P=0.24 for RRM1), or between gene expression and progression-free survival (HR, 1.00 and P=0.23 for ERCC1; HR, 0.998 and P=0.36 for RRM1), as would be expected for a study of this design and size. Patients assigned to each of the four treatment groups demonstrated similar outcomes (P=0.98 for overall survival; P=0.58 for progression-free survival), which suggests that using ERCC1 status to select treatment may improve patient outcomes in patients resistant to platinum-based chemotherapy[48].
- A phase III trial investigated whether selecting patients for cisplatin treatment based on pretreatment ERCC1 levels in tumor specimens improved patient outcomes. Of 444 patients with stage IIIb or stage IV NSCLC, 346 were evaluable for response and participated in this multicenter, randomized study. Patients were randomly assigned in a 1:2 ratio to either the control arm or the genotypic arm. All patients in the control arm received cisplatin in combination with docetaxel. Patients in the genotypic arm were treated based on ERCC1 mRNA expression levels: those with low levels received cisplatin in combination with docetaxel, while those with high levels received docetaxel in combination with gemcitabine. All patients were treated with six cycles of chemotherapy or until disease progression, death, or a serious adverse event occurred. When comparing treatment arms, it was found that overall response rate was higher in the genotypic group than in the control group (51.2% versus 39.3%; P=0.02). Moreover, univariate logistic regression models indicated that the probability of an objective response was 1.59 times (95% CI, 1.03 to 2.47 times) greater for those in the genotypic arm when compared with those in the control arm (P=0.04). When comparing the two groups in the genotypic arm (high versus low ERCC1 levels), there was no significant difference in overall response rate (43.8% versus 53.2%; P>0.05). Median overall survival was not significantly different between treatment arms (9.8 months for control arm versus 9.9 months for the genotypic arm; P=0.59). Median progression-free survival was also not significantly different between the two groups (5.2 months for control arm versus 6.1 months for genotypic arm; P=0.30)[49].

## **Limitations**

- Methodology - different studies used different methodologies (i.e. IHC versus RT-PCR, the implications of which is unclear when reviewing the body of evidence. In addition, not all studies use the same method to establish a “cut-off” value for distinguishing high and low levels of *ERCC1* expression; thus, it is not clear that different studies are directly comparable. Finally, in the absence of optimization and standardization of ERCC1 expression data, the subjectivity of pathological examination and the heterogeneity of staining may impact test interpretation.
- Patient populations - most studies involved relatively small numbers of patients (< 100 patients per study) and the patient populations included tended to be heterogeneous in nature (subjects had been treated with a variety of chemotherapy regimens and for a variable number of treatment cycles, had different stages of NSCLC, and had different different tumor types (i.e. adenocarcinoma versus squamous cell carcinoma), which limits the generalizability of results.

## **Conclusions**

The body of evidence surrounding ERCC1 testing in NSCLC indicates that the analytical validity of ERCC1 testing using IHC may have limitations. The results of one study questioned the specificity of the 8F1 antibody for the ERCC1 protein[21].Two studies found some disconcordance between primary and secondary tumors with regards to ERCC1 expression levels[23] [24], which raises the possibility that analysis of the primary tumor may lead to an incorrect approach for a metastatic tumor[24]. However, it should be noted that the relevance of the results of these studies to the commercially available assays is not clear. 

As a prognostic marker, the results of 5 of 7 studies would suggest that patients with high levels of expression of the ERCC1 protein generally have a longer survival time than patients with low levels of expression of the protein. In contrast, as a predictive marker in treatment with platinum-based chemotherapies, the results of 11 of 17 studies indicated that patients with low levels of expression of the ERCC1 protein typically have a longer survival time than patients with high expression levels of the protein. This apparent contradiction may be explained if high levels of expression of ERCC1 are causing resistance to platinum-based chemotherapy, which has been observed in *in vitro* studies[10]. While the available clinical validity data suggest a relationship between ERCC1 and responce to platinum-based chemotherapies, are some inconsistencies in the data, the number of patients tested is small and the data are retrospective.

A prospective phase III clinical utility study with 346 evaluable patients has examined if selecting patients for cisplatin treatment could improve patient outcomes[48]. Patients were randomized to standard therapy with cisplatin plus docetaxol, or to a genotypic arm. In the genotypic arm, patients with low levels of expression of ERCC1 received cisplatin plus docetaxol, but patients with high levels of ERCC1 expression received docetaxol plus gemcitabine. A significantly higher response rate for patients in the genotypic arm with low levels of expression of ERCC1 was observed than patients in the control arm who received the same regimen of cisplatin plus docetaxol. However, there was no significant difference in response rates between the patients with low levels of expression that received cisplatin plus docetaxol and those with high levels of expression of ERCC1 that received docetaxol plus gemcitabine. Nor were there differences in progression-free survival or overall survival rates amongst the groups. Interestingly peripheral neurotoxicity occured significantly more often in pateints in the genotypic arm with low levels of expression of ERCC1[48] . While this prospective clinical utility study confirms the relationship between cisplatin treatment and ERCC1 expression, it fails to demonstrate that switching patients with high expression levels of ERCC1 from cisplatin to another treatment (gemocitabine) improves patient outcomes as the study design did not include an appropriate control and the choice of gemcitabine as the switching agent may not have been optimal because of its interaction with the NER pathway[50].

Overall the available evidence indicates that there is a potential for ERCC1 testing to aid in selecting a chemotherapy regimen and thereby to improve patient outcomes. However, there are shortcomings with regards to the analytical validity data on the commercially available ERCC1 assays, the data on clinical valdity is retrospective in nature from mostly small studies. Finally there are no prospective studies of clinical utility that have demonstrated that switching treatment regimens based on ERCC1 expression levels leads to improvements in patients outcomes. Given the inconsistencies in the data and the lack of demonstrable clinical utility, current evidence would likely prove insufficient to support the routine use of this test in the care of patients with NSCLC. 

## **Links**

- **ClinicalTrials.gov:** ERCC1 and NSCLC. A total of 10 open studies are listed in this search for "ERCC1" and "non-small cell lung cancer". All of these studies are designed to evaluate the relationship between ERCC1 status and the response to various treatment regimens. At least 5 of these studies examine additional biomarkers, such as expression of the *RRM1 *and breast cancer 1 (BRCA1) genes[50].
- **Online Mendelian Inheritance in Man:** ERCC1.
- **U.S. Food and Drug Administration:** Table of Valid Genomic Biomarkers in the Context of Approved Drug Labels

Last updated: *[indicate month, day, and year of publication of most recent version]*

## **Acknowledgments**

The authors would like to acknowledge the contributions of the members of the Hayes Genetic Test Evaluation team, particularly Lisa Spock, Linnie Wieselquist and Charlotte Kuo-Benitez.

## **Funding information**

Funding for the Health Technology Assessment that informed this work was provided by Hayes, Incorporated. Funding to create this Knol was provided by the Centers for Disease Control and Prevention under Contract No. 200-2009-F-32675. This funding was distributed through the Genetic Alliance.  

## **Competing interests**

The authors are employees at Hayes, Inc., an independent health technology research and consulting company. None of the employees at this company has any financial or personal interest in any of the technologies reviewed by Hayes, Inc.. No input on report content or conclusions is permitted by manufacturers. Although the CDC funded the work to produce this article, the content is based entirely on Hayes, Inc.'s own analysis and there was no input from the CDC.
